# Papain ameliorates monocyte-platelet aggregate formation-mediated inflammatory responses in monocytes by upregulating miRNA-146a transcription

**DOI:** 10.1371/journal.pone.0278059

**Published:** 2022-11-21

**Authors:** Lei Jiang, Chan Xu, Yan Zhao, Qinghua Huang, Wufeng Yuan, Yan Wu, Xianming Fei

**Affiliations:** 1 Laboratory Medicine Center, Department of Clinical Laboratory, Zhejiang Provincial People’s Hospital, Affiliated People’s Hospital, Hangzhou Medical College, Hangzhou, Zhejiang, China; 2 Department of Laboratory Medicine, Third Affiliated Hospital of Zhejiang Chinese Medical University, Hangzhou, Zhejiang, China; 3 Heart Center, Department of Cardiovascular Medicine, Zhejiang Provincial People’s Hospital, Affiliated People’s Hospital, Hangzhou Medical College, Hangzhou, Zhejiang, China; 4 Geriatric Medicine Center, Department of Endocrinology, Zhejiang Provincial People’s Hospital, Affiliated People’s Hospital, Hangzhou Medical College, Hangzhou, Zhejiang, China; 5 Department of Laboratory Medicine, Lin’an First People’s Hospital of Hangzhou, Hangzhou, Zhejiang, China; Xiangtan University, CHINA

## Abstract

**Background:**

MicroRNA-146a (miRNA-146a) is a nuclear factor κB (NF-κB)-inducible and inflammation-sensitive miRNA, while papain elicits anti-inflammatory effects by inhibiting monocyte-platelet aggregate (MPA)-mediated NF-κB pathway activation in monocytes. This study aimed to demonstrate the underlying effects of papain on MPA formation-initiated miRNA-146a expression and subsequent action in monocytes.

**Methods:**

THP-1 cells were exposed to papain, miRNA-146a mimic and inhibitor, NF-κB inhibitor (BAY11-7082), and platelets. Flow cytometry was used to measure the MPA formation-initiated monocyte activation. Levels of miRNA-146a, cyclooxygenase 2 (COX-2) mRNA and protein, and monocyte chemoattractant protein 1 (MCP-1) were analyzed in monocytes by RT-PCR, western blot, and ELISA.

**Results:**

The NF-κB inhibitor and miRNA-146a mimics upregulated miRNA-146a expression but suppressed subsequent monocyte activation and expression of COX-2 and MCP-1. Following exposure to papain, the enhanced miRNA-146a transcription induced by MPA-formation was found along with significant inhibition of monocyte activation in a dose-dependent manner. However, the inhibitory tendency was significantly reversed by miRNA-146a inhibitors. Expression of COX-2 mRNA and protein, as well as MCP-1, was inhibited in monocytes by papain, whereas miRNA-146a inhibitors promoted COX-2 and MCP-1 expression.

**Conclusion:**

Our findings suggest that papain can inhibit MPA formation-mediated expression of inflammatory mediators in activated monocytes by upregulating miRNA-146a transcription.

## Introduction

Atherosclerotic cardiovascular disease presently accounts for the majority of mortality worldwide. Atherosclerosis (AS), a chronic arterial disease, is the leading cause of vascular death [1]. Patients with AS will display no symptoms at the beginning, but AS can gradually progress into coronary artery disease [2]. Many studies have demonstrated a strong association between inflammation and the risk of AS and future cardiovascular events. During the development and progression of AS, monocytes play a central role in the vascular endothelium [3], whereby they promote and participate in the inflammation process by releasing proinflammatory cytokines [4]. A previous study demonstrated that the formation of monocyte-platelet aggregates (MPAs) induces monocyte activation and release of proinflammatory cytokines, subsequently facilitating the development of AS [5]. MPAs are early markers of subclinical AS [6] and a useful indicator of platelet activation [7]. Moreover, increased levels of MPAs are present in acute coronary syndrome [8]. Thus, identifying the mechanisms underlying monocyte activation by MPA formation and inhibitors of activated monocytes would be of great clinical significance for the prevention and treatment of atherosclerotic diseases.

Activated platelets preferentially bind to circulating monocytes and form monocyte-platelet complexes with increased adhesive and migratory properties [8]. Some of the mechanisms involved include nuclear factor κB (NF-κB) pathway activation and increased secretion of proinflammatory mediators [9], such as monocyte chemoattractant protein 1 (MCP-1) [10]. Under proinflammatory conditions, the enhancement of MPAs subsequently expands the pool of circulating monocytes in a cyclooxygenase-2 (COX-2) dependent manner [11]. Dixon et al. reported that binding of P-selectin on the surface of platelet to P-selectin glycoprotein ligand of monocyet can trigger outside-in signaling leading to NF-κB activation and COX-2 transcription [12]. Substantial evidence indicates that unregulated COX-2 expression and prostaglandin synthesis influence chronic inflammatory conditions, including AS and its complications [13]. Thus, the binding of activated platelets to monocytes and subsequent monocyte activation may mediate AS progression by integrating multiple inflammatory signaling pathways [14]. Indeed, interruption of signaling pathways that activate monocytes, such as the NF-κB/COX-2 pathway triggered by adherence of platelets to monocytes, may be a new target for molecular intervention [4]. Our previous study revealed that monocyte COX-2 expression was closely associated with MPA formation and MCP-1 levels in patients with coronary artery disease [15]. Therefore, enhanced COX-2 expression might play a key role in inflammatory processes related to MPAs formation-mediated monocyte activation, which may be ameliorated by inhibiting the NF-κB signaling pathway upstream of COX-2 expression initiated by MPAs formation.

MicroRNAs (miRNAs or miRs) are small noncoding RNAs that posttranscriptionally regulate gene expression [16]. Emerging studies indicate that tissue repair and injury, as well as immune and inflammatory responses, are substantially regulated by miRNAs. In particular, monocytes/macrophages are critical for mounting and resolving inflammatory responses during tissue injury and repair [17]. miRNA-146, especially miRNA-146a, is one the first recognized miRNAs associated with activation of the NF-κB activation and constitutes a negative feedback loop, whereby NF-κB activates miRNA-146 to suppress NF-κB signaling [18]. Lin et al. reported that, in hyperlipidemic mice, treatment with miRNA-146a mimics reduced Ly-6C^high^ monocytosis, lesional macrophage numbers, macrophage inflammatory responses, and AS [19], indicating that upregulation of miR146a expression could be a therapeutic strategy for inflammation mediated by the NF-κB signaling pathway in monocytes/macrophages.

Papain, a member of the cysteine protease enzyme family, might be as effective as bromelain in preventing platelet aggregation and reducing platelet adhesion to endothelial cells on the basis of mass concentrations [20]. Our previous studies revealed that papain elicits protective effects against MPA formation and subsequent monocyte activation. In addition, papain induced inflammatory responses by inhibiting COX-2 expression through regulation of the ERK/p38 and PI3K/Akt pathways in THP-1 cells [21], suggesting it is an effective inhibitor of the NF-κB signaling pathway.

Our previous studies revealed papain to be a likely intervening agent in MPA-mediated inflammatory processes through upstream inhibition of the NF-κB signaling pathway in monocytes. However, whether papain can regulate miRNA-146a transcription to reduce inflammatory responses resulting from NF-κB signaling activation mediated by MPA formation remains unclear. In this study, we aimed to explore the inhibitory mechanisms of papain on expression of inflammatory mediators initiated by MPA formation via regulation of miRNA-146a transcription in THP-1 cells, and further elucidate the mechanisms regulating this action.

## Materials & methods

### Participants

In this study, five healthy volunteers were recruited in August 2020, including three men and two women aged from 23 to 30 years. No subjects had taken any medicines for 1 week before sample collection. Approximately 25 mL of peripheral blood were collected in lithium heparin-containing tubes from them volunteers the morning after an overnight fast. Written informed consent was obtained from all subjects. This study was approved by the ethics committee of Zhejiang Provincial People’s Hospital (Approval No. 2019KY124).

### Platelet isolation

The method for platelet isolation was optimized based on our previous description [21]. In brief, blood samples were centrifuged at 150 × *g* for 8 min to isolate the upper layer containing platelet-rich plasma, and then gently mixed with 1.0 μmol/L prostaglandin E1. Subsequently, the mixture was placed for 5 min at 18°–25°C and centrifuged at 500 × *g* for 5 min. The resulting pellet was resuspended, washed once with normal saline solution, and centrifuged at 500 × *g* for 5 min. Next, the resulting pellet was resuspended with Tyrode’s buffer. After counting with an automatic hematology analyzer (Mindray, BC-6900), platelets were adjusted to a solution with containing 2 × 10^10^ platelets/L for subsequent experiments.

### THP-1 cell culture and transfection

#### THP-1 cell culture

THP-1 cell is a cell line of acute monocyte leukemia, and it is an ideal tool for studying immunity and inflammation, and is widely used in various research of monocyte/macrophage-related mechanisms. In this study, human THP-1 cells were obtained from Sai Baikang Biotechnology (Shanghai, China) and cultured in RPMI-1640 medium (SH30809.01; HyClone, Logan, UT) containing 10% fetal bovine serum (FBS; SH30256.01, HyClone). Cells were prepared as a suspension (5 × 10^7^ cells/L) and incubated at 37°C in a humidified atmosphere containing 5% CO_2_ for subsequent experiments.

#### Transfection regent preparation

MiRNA-146 mimic and inhibitor sequences (RNA oligos, including negative controls) were synthesized by GenePharma Company (Suzhou, China) according to the sequences presented in **Table 1.** Each RNA oligo was centrifuged at 1500 × g for 3 min and then dissolved in 30 μL of DEPC solution (1 OD) at 1 μg/mL. For transfection, 5 μL of RNA oligo solution in 120 μL of Opti-MEM™ Medium (Invitrogen, Carlsbad, CA) and 7.5 μL of Lipofectamine® 3000 (Invitrogen) solution in 117.5 μL of medium were gently mixed and incubated at room temperature for 5 min. Finally, the two solutions were gently mixed to form a complex and incubated at room temperature for 20 min.

**Table 1:** Sequences of miRNA-146a mimics and inhibitors.

Gene	Forward Primer	Reverse Primer
miRNA-146a mimics	UGAGAACUGAAUUCCAUGGGUU	CCCAUGGAAUUCAGUUCUCAUU
miRNA-146a mimics NC	UUCUCCGAACGUGUCACGUTT	ACGUGACACGUUCGGAGAATT
miRNA-146a inhibitors	AACCCAUGGAAUUCAGUUCUCA	
miRNA-146a inhibitors NC	CAGUACUUUUGUGUAGUACAA	

#### Cell transfection

After aspirating and discarding the medium in wells containing THP-1 cell cultures, the wells were washed with fresh medium and 750 μL of medium was added along with 250 μL of the transfection complex solution. After incubation for 6 h, the medium was discarded and 2 mL of complete medium with 10% FBS was added into the wells containing THP-1 cells for 48 h. Next, real-time PCR was used to evaluate expression levels of miRNA-146a after transfection. High expression of miRNA-146a in mimic-transfected cells and low expression in inhibitor- and negative control-transfected cells was considered a successful transfection. Detailed methods are described in **Real-time polymerase chain reaction** section below.

### Transfected cell cultures and treatments

#### Effect of miRNA-146a on MPA-mediated monocyte activation

A total of 75 μL of transfected THP-1 cell suspension was added into 75 μL of platelet suspension and mixed gently. Each group was treated with 100 U/L of thrombin (final concentration; Siemens Healthcare Diagnostics Products, Erlangen, Germany). To observe the effect of NF-kB inhibitors on miRNA-146a expression, BAY11-7082 (1 μmol/L final concentration) was added into the co-culture system (with miRNA-146a mimic negative controls and inhibitor negative controls, but without miRNA-146a mimic or inhibitor) in the presence of 100 U/L of thrombin. An additional blank control group contained a mixture of THP-1 cells, platelets, and saline. Mixtures for each group were incubated at 37°C for 2 h and prepared for subsequent miRNA-146a detection and related assays.

#### Effect of papain on MPA-mediated miRNA-146a expression

Human THP-1 cells transfected with miRNA-146a inhibitors or negative controls were treated with papain solution as follows: blank group (miRNA-146a negative controls with saline), papain groups (miRNA-146a negative controls with 100 or 200 U/L papain), and inhibitor groups (miRNA-146a inhibitors with 100 or 200 U/L papain). Subsequently, 75 μL of platelet suspension and 100 U/L of thrombin (final concentration) were added into 75 μL of treated THP-1 cell mixture and gently mixed. Mixtures for all groups were incubated at 37°C for 2 h and used to perform miRNA-146a detection and related assays.

#### Real-time polymerase chain reaction (RT-PCR)

Total RNA from samples was isolated with Trizol reagent according to the manufacturer’s protocol (Sangon BioTech, Shanghai, China). One microgram (1 μg) of total RNA was reverse transcribed into cDNA using SuperRT cDNA Synthesis Kit cDNA (CW2569; CwBiotech, Beijing, China). Expression levels of miRNA-146a and COX-2 genes were evaluated by performing RT-PCR (Bio-Rad, Hercules, CA) analysis utilizing UltraSYBR Premix Ex TaqII Mixture (High ROX) (RR820A; Takara, Kusatsu, Japan). Primer sequences for miRNA-146a and COX-2 are presented in **Table 2**. Human U6 and GAPDH served as internal controls. Relative expression of miRNA-146a and COX-2 genes was calculated according to the 2^-ΔΔCt^ method.

**Table 2:** Primer Sequences of human miRNA-146a and COX-2.

Gene	Forward Primer	Reverse Primer
Human miRNA-146a	CAGTGCGTGTCGTGGAGT	GGGTGAGAACTGAATTCCA
Human U6	AAAGCAAATCATCGGACGACC	GTACAACACATTGTTTCCTCGGA
Human COX-2	ACTACCCCATGCCAGAAGAG	TCATTGGAGCGACGGTTCATC
Human GAPDH	GGAGCGAGATCCCTCCAAAAT	GGCTGTTGTCATACTTCTCATGG

#### Flow cytometry (FCM)

Treated THP-1 cells that adhered to the plate wall were digested with trypsinase, collected, and washed twice with phosphate-buffered saline (PBS). Next, cells were resuspended with 300 μL of PBS. For measurements of monocyte activation mediated by MPAs, mixtures for each group were incubated with 10 μL of FITC anti-human CD11b antibody (mouse anti-human monoclonal; 301329; Biolegend, San Diego, CA) and 3 μL isotype-matched controls for 30 min in darkness at room temperature. After incubation, cells were washed twice with PBS to dispose of unbound antibodies, and resuspended in 300 μL of PBS. Subsequently, viable CD11b positive (CD11b^+^) monocytes were analyzed and counted by FCM (C6 BD Accuri; BD Biosciences, Franklin Lakes, NJ). After performing FCM, activated monocyte events were gated by identifying CD11b^+^ cells.

#### Western blot analysis

In brief, samples were centrifuged and the resulting pellet was resuspended in RIPA cell lysis solution containing PMSF and protease inhibitors (Beyotime Biotech, Haimen, China). After shaking vigorously, cells lysed on ice for 30 min. Subsequently, the lysis solution was centrifuged at 12,000 × *g* for 5 min at 4°C. The supernatant was collected and transferred to a new pre-cooled centrifuge tube. A quantitative protein analysis was performed with a bicinchoninic acid kit (Solarbio, Beijing, China) according to the manufacturer’s instructions. To perform sodium dodecyl sulfide polyacrylamide gel electrophoresis (SDS-PAGE), a total of 30 μg protein was used. COX-2 protein was detected using an anti-COX-2 primary antibody (rabbit anti-human monoclonal, Cox2 (D5H5) XP®, 12282T) purchased from Cell Signaling Technology (Danvers, MA). A GAPDH antibody (rabbit anti-human monoclonal, AF7021) was purchased from Affinity Biotechnology (Jiangsu, China). Relative protein analysis was processed based on Quantity One software (Bio-Rad).

#### Enzyme-linked immunosorbent assay (ELISA)

In each co-incubated group, the cell suspension was diluted with PBS (pH7.2–7.4) to adjust the cell density to 1 × 10^6^ cells/mL. Next, repeated freezing and thawing was performed to destroy the cell membranes and release intracellular components. After the samples were centrifuged for 20 min (1500–2000 × *g*) at room temperature, the supernatant was collected. Synthesis levels of MCP-1 in THP-1 cells were determined by ELISA (MM-0081H2; MeiMian, Chengdu, China) according to the manufacturer’s instructions. Briefly, 25 μL of samples, 50 μL of standard, and 25 μL of diluent were added into the wells of an ELISA plate (except for the blank well,) and 100 μL of MCP-1 monoclonal antibody labeled with horseradish peroxidase was added for incubation at 37°C for 60 min. After washing five times with cleaning solution, 100 μL of the chromogenic substrate Tetramethylbenzidine (TMB) was added and incubated for 15 min at 37°C in darkness. After terminating the reaction, the optical density value was measured at a 450-nm wavelength by a microplate reader (CMaxPlus; Molecular Devices, San Jose, CA). Finally, the level of MCP-1 was calculated based on a standard curve.

#### Statistical analysis

Each experiment was independently performed at least three times. Data were first tested for distribution normality by the Kolmogorov-Smirnov test (K-S test), and normally distributed data are presented as mean ± standard deviation (mean ± SD). Relative protein levels were measured using Quantity One software. One-way analysis of variance (ANOVA) was adopted for comparisons among multiple groups, while Fisher’s least significant difference (LSD) t-test was used to compare differences between two groups. All statistical analyses were performed with SPSS software (version 25.0; Chicago, IL). P values less than 0.05 were considered statistically significant.

## Results

### NF-κB inhibition upregulates miRNA-146a expression in monocytes activated by MPAs formation

**Fig 1:**
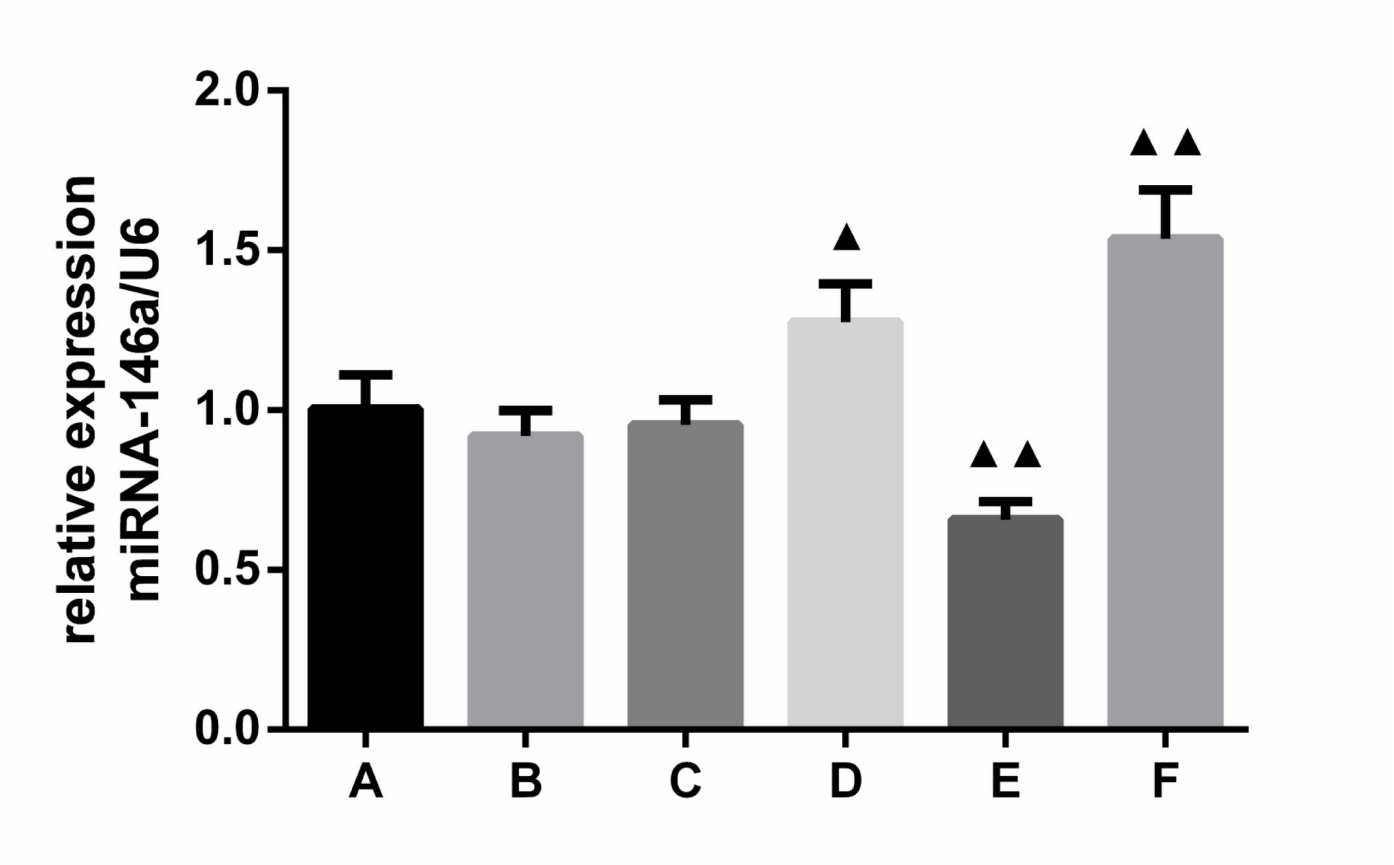
Expression levels of miRNA-146a in THP-1 cells activated by MPA formation ($\bar{x}$ ± *s*, *n* = 3). A: negative control; B: miRNA-146a mimics NC; C: miRNA-146a inhibitors NC; D: NF-κ inhibitors; E: miRNA-146a inhibitors; F: miRNA-146a mimics; NC: negative control; MPAs: monocyte-platelet aggregates; ^▲^*P* < 0.05 compared with negative control group,^▲▲^*P* < 0.01 compared with miRNA-146a inhibitor and mimic NC groups, as analyzed by LSD-*t* test.

Given that MPA formation increases under activated platelet/monocyte attachment, to determine the effects of NF-κB signaling inhibition on miRNA-146a expression of monocytes activated by MPA formation, we co-cultured miRNA-146a-transfected THP-1 cells and activated platelets induced by thrombin. In addition, an NF-κB inhibitor was added to THP-1 cells to observe the effects on miRNA-146a expression in activated monocytes. After performing RT-PCR with primers for miRNA-146a, we found that, compared with controls, miRNA-146a mRNA expression was significantly increased (P < 0.05 or P < 0.01) in both BAY11-7082 (1.276 ± 0.120 *vs*. 1.004 ± 0.107; p = 0.043) and miRNA-146a mimic (1.537 ± 0.152 *vs*. 0.918 ±0 .080; p = 0.008) groups. In contrast, miRNA-146a mRNA expression was decreased in the miRNA-146a inhibitors group (0.657 ± 0.056 vs. 0.953 ± 0.548) (p = 0.008) (**Fig 1)**. These results demonstrate that administration of an NF-κB inhibitor elicited similar effects to miRNA-146a mimics on expression of miRNA-146a in monocytes activated by MPA formation.

### MiRNA-146a inhibits MPA formation-evoked monocyte activation

**Fig 2:**
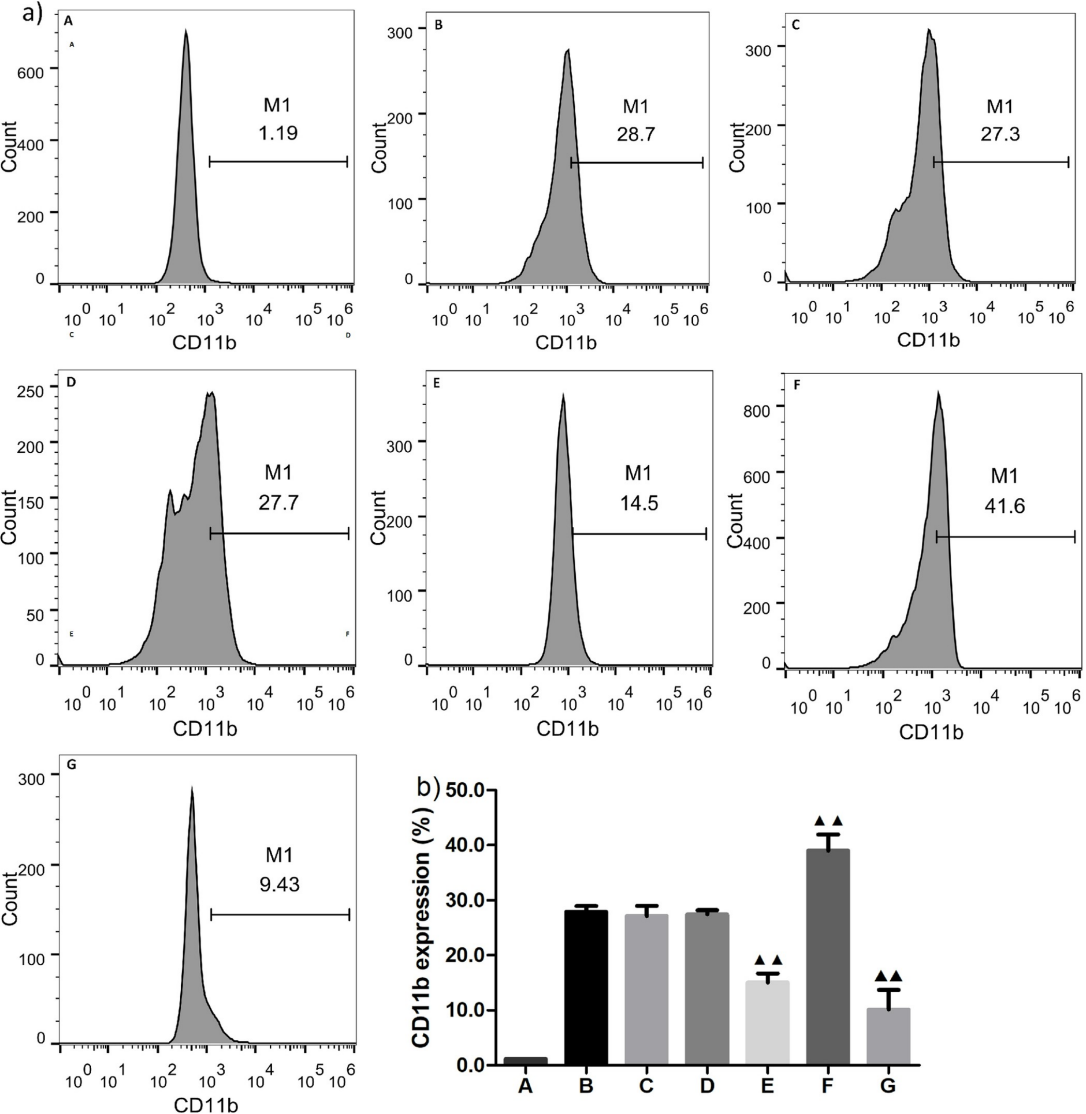
Effects of miRNA-146a on MPA-mediated CD11b expression in activated monocytes. ($\bar{x}$ **± *s*, *n* = 3**).a) Gating strategy for quantification of MAC-1 in a co-culture system of THP-1 cells and activated platelets by flow cytometry (FCM). MAC-1 was evaluated by expression of CD11b in THP-1 cells following mediation by monocyte-platelet attachment under different treatment as follows: A: isotype control; B: negative control; C: miRNA-146a mimics NC; D: miRNA-146a inhibitors NC; E: BAY11-7082; F: miRNA-146a inhbitors; G: miRNA-146*a mimics*. *NC*: *negative control*. ^*▲*^*P < 0*.*05 compared with miRNA-146a inhibitors NC group*, ^*▲▲*^*P* < 0.01 compared with negative control, miRNA-146a inhibitor, and mimic NC groups, as analyzed by LSD-t test.

Our previous study indicated that monocyte-platelet attachment (MPA formation) increases expression of the surface marker Mac-1 (CD11b) on activated monocytes (*Fei XM*, *et al*., *2018*). Thus, to further confirm the inhibitory effects of miRNA-146a on MPA formation-induced monocyte activation, we evaluated MPA-mediated expression levels of Mac-1 (CD11b) in the presence of thrombin, miRNA-146a mimics, miRNA-146a inhibitors, and the NF-κB inhibitor BAY11-7082 with FCM, respectively. FCM results show that CD11b expression levels were markedly decreased following treatment with BAY11-7082 or miRNA-146a mimics (15.07% ± 1.63% vs. 27.90% ± 1.06%, p = 0.000; and 10.16% ± 3.54% vs. 27.17% ± 1.80%, p = 0.001, respectively), and increased by miRNA-146a inhibitors (37.75% ± 2.63% vs. 28.43% ± 0.74%; p = 0.000) (**Fig 2A and 2B**). From these results, we concluded that miRNA-146a might have remarkable inhibitory effects on MPA formation-induced activation of monocytes, probably through inhibition of the NF-κB signaling pathway, and its inhibitors can reverse these effects.

### MPA-mediated inflammatory responses involving activated monocytes are inhibited by miRNA-146a

**Fig 3:**
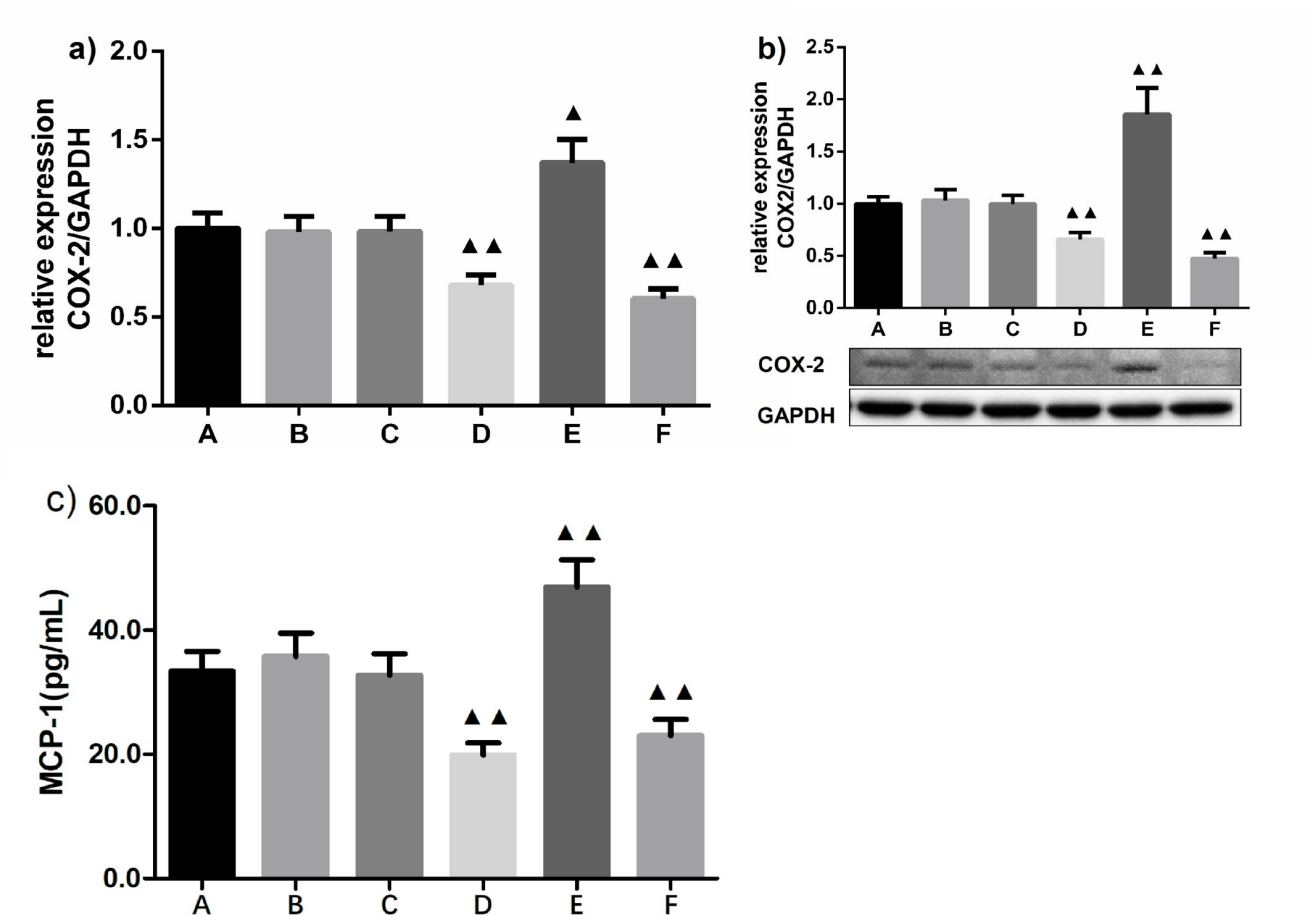
Effect of miRNA-146a on COX-2 expression and MCP-1 secretion of MPA-mediated THP-1 cells ($\bar{x}$ ± *s*, *n* = 3 for COX-2 and *n* = 6 for MCP-1). a) COX-2 transcriptional levels detected by RT-PCR; b) COX-2 protein expression analyzed by western blotting, and c) MCP-1 expression measured by ELISA. A: negative control; B: miRNA-146a mimics NC; C: miRNA-146a inhibitors NC; D: BAY11-7082; E: miRNA-146a inhibitors; F: miRNA-146a mimics; NC: negative control; COX-2: cyclooxygenase-2; ^▲^*P* < 0.05 compared with miRNA-146a inhibitors NC group, ^▲▲^*P* < 0.01 compared with negative control, miRNA-146a inhibitor, and mimics NC groups, as analyzed by LSD-*t* test.

To observe the effect of miRNA-146a expression on inflammatory responses involved in monocyte activation, we evaluated the expression of downstream inflammatory mediators potentially related to AS. Given that levels of COX-2 and MCP-1 are indicators of monocyte activation in many diseases, such as cardiovascular disease, we analyzed expression levels of COX-2 mRNA and protein, as well as MCP-1, in monocytes activated by MPA formation. The results of RT-PCR and western blotting showed that both transcription and protein expression levels of COX-2 were markedly decreased in the presence of miRNA-146a mimics and the NF-κB inhibitor BAY11-7082, but increased by miRNA-146a inhibitors (**Fig 3A and 3B**). Moreover, MCP-1 levels exhibited similar tendencies to COX-2 expression **(Fig 3C)**. These findings indicate that both miRNA-146a and the NF-κB inhibitor effectively inhibited MPA formation-mediated expression of COX-2 and MCP-1 in activated monocytes, and miRNA-146a inhibitors can reverse the protective effects of miRNA-146a on monocyte activation. These results also reveal a potential mechanism by which miRNA-146a suppresses the NF-κB signaling pathway.

### MiRNA-146a inhibitors reverse the inhibitory effects of papain on MPA formation-initiated monocyte activation

**Fig 4:**
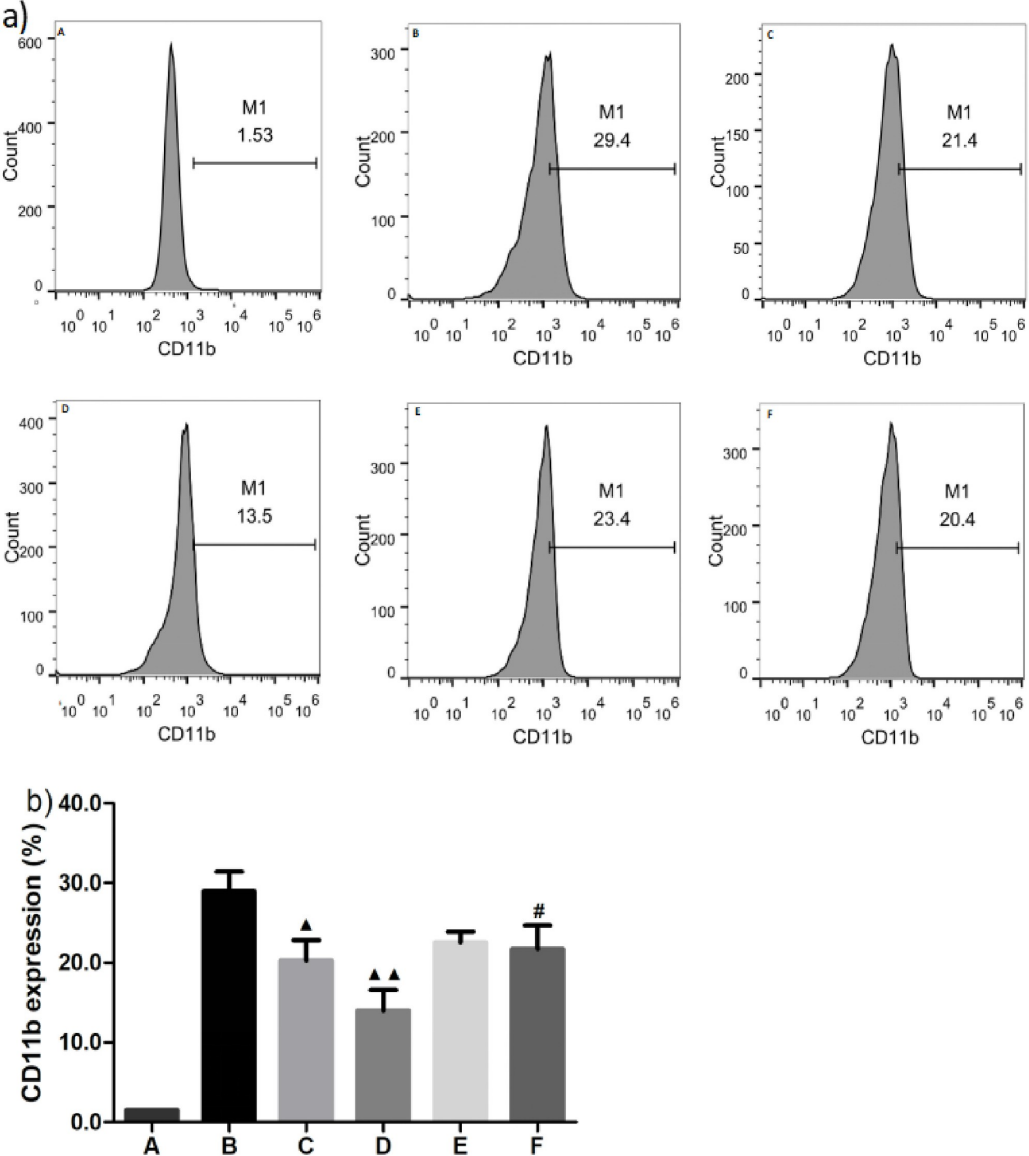
Effects of papain and miRNA-146a inhibitors on CD11b expression of activated monocytes mediated by MPA formation. ($\bar{x}$ ± *s*, *n* = 3). a) Gating strategy for quantification of Mac-1 in a co-culture system of THP-1 cells and activated platelets by flow cytometry (FCM). Mac-1 was evaluated by expression of CD11b in THP-1 cells mediated by monocyte-platelet attachment following treatment with 100 or 200 U/L of papain with or without miRNA-146a. A: isotype control; B: negative control; C: 100 U/L of papain; D: 200 U/L of papain; E: 100 U/L of papain combined miRNA-146a inhibitors; F: 200 U/L of papain combined miRNA-146a inhbitors. ^**▲, #**^*P* < 0.05 compared with negative control and 200 U/L papain group, and ^**▲▲**^P < 0.01 compared with negative control, as analyzed by LSD-*t* test.

The above results revealed the effects of miRNA-146a mimics and inhibitors, as well as an NF-κB inhibitor, on MPA-mediated monocyte activation and related expression of inflammatory cytokines in monocytes. To observe the effects of papain and miRNA-146a on monocyte activation, 100 and 200 U/L of papain solution and miRNA-146a inhibitors were used to treat THP-1 cells in a co-culture system. It has been recognized that THP-1 cell lines were the monocyte-derived cells, therefore, we only defined the CD11b^+^ THP-1 cells as the activated monocytes and measured the CD11b expressions on the surface of the cells. In this study, we discovered that MPA-mediated upregulation of activated monocytes (CD11b^+^ cells) was effectively inhibited by both 100 and 200 U/L of papain (20.30% ± 2.54% and 14.03% ± 2.54% *vs*. 28.97% ± 2.48%; p = 0.013 or 0.002, respectively) in the co-culture system of THP-1 cells and activated platelets (**Fig 4A–4C** and **4A–4D** vs. **4A–4B**, **third** and fourth columns vs. second column in **Fig 4B**, respectively). miRNA-146a inhibitors reverse the protective effects of papain (22.15% ± 1.63% vs. 20.30% ± 2.54%, and 22.40% ± 3.82% vs. 14.03% ± 2.54%, p = 0.026 for 200 U/L of papain) (**Fig 4A–4E** and **4A–4F** vs. **4A–4C** and **4A–4D**, **fifth and sixth** columns vs. third and fourth columns in **Fig 4B**, respectively), further indicating that papain is a potentially powerful inhibitor of MPA-mediated monocyte activation, while miRNA-146a inhibitors can reverse the powerful blocking effect of papain on monocyte activation initiated by MPA formation.

### MPA-mediated COX-2 mRNA expression is inhibited in the presence of papain by upregulation of miRNA-146a transcription

**Fig 5:**
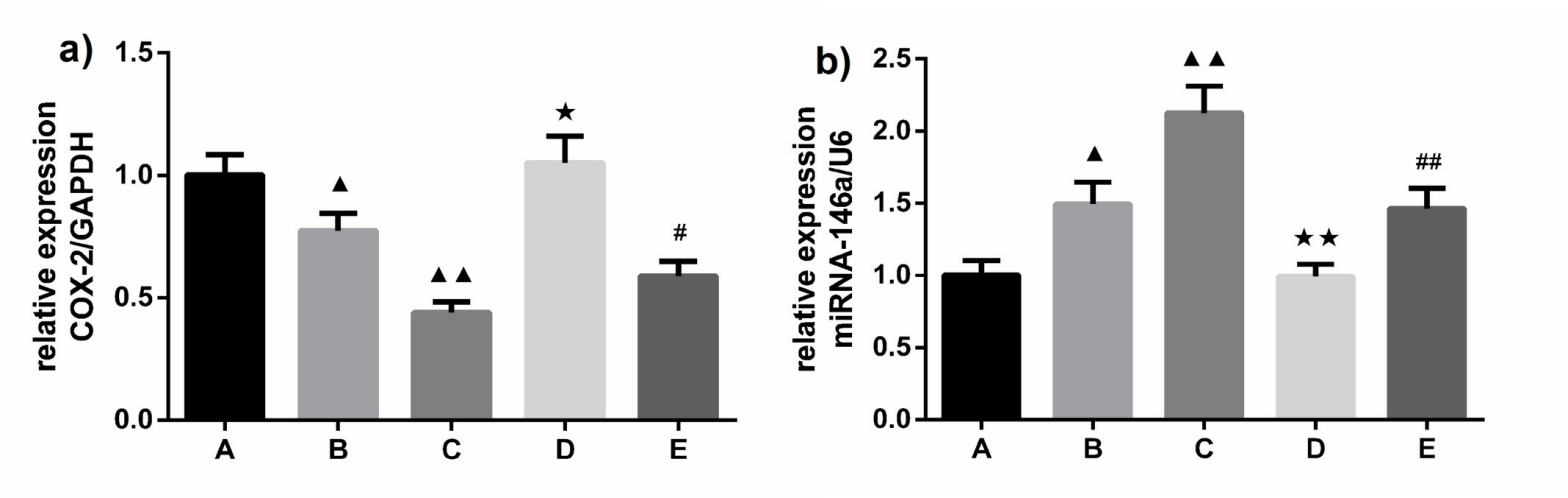
Effects of varying papain concentrations on MPA-mediated expression of COX-2 mRNA and miRNA-146a. ($\bar{x}$ ± *s*, *n* = 3). RT-PCR was used to detect expression of a) COX-2 mRNA and b) miRNA-146a in a co-culture system of THP-1 cells and activated platelets following 100 or 200 U/L of papain treatment. A: negative control; B: 100 U/L papain; C: 200 U/L papain; D: 100 U/L papain combined miRNA-146a inhibitor; E: 200 U/L papain combined miRNA-146a inhibitors. ^**▲, ✭, #**^*P* < 0.05 and ^**▲▲, ✭✭, ##**^*P* < 0.01 compared with negative control, 100 U/L papain, and 200 U/L papain groups, respectively, as analyzed by LSD-*t* test.

As the above-mentioned results show, increased monocyte COX-2 expression represents an enhanced response of inflammation related to activated monocytes, which can be upregulated by an miRNA-146a inhibitor and inhibited by miRNA-146a or an NF-κB inhibitor. In this study, we first observed the effects of different concentrations of papain solution on mRNA expression of miRNA-146a and COX-2 activated by platelet-monocyte attachment. Our findings show that the MPA-mediated upregulation of COX-2 mRNA expression was effectively inhibited by 100 and 200U/L of papain (0.774 ± 0.071 and 0.440 ± 0.043 *vs*. 1.002 ± 0.082; p = 0.022 and 0.000, respectively), and an miRNA-146a inhibitor can reverse the protective effects of papain (1.052 ± 0.107 *vs*. 0.774 ± 0.071 and 0.589 ± 0.060 *vs*. 0.440 ± 0.043; p = 0.020 and 0.025, respectively) (**Fig 5A).** Further results revealed that miRNA-146a expression was upregulated by 100 and 200U/L of papain (1.494 ± 0.154 and 2.126 ± 0.186 *vs*. 1.003 ± 0.099; p = 0.010 and 0.000, respectively), and miRNA-146a inhibitors can reverse the facilitating effects of 100 and 200U/L of papain on miRNA-146a expression (0.992 ± 0.087 *vs*. 1.494 ± 0.154 and 1.464 ± 0.186 vs. 2.126 ± 0.186; p = 0.008 and 0.008, respectively) **(Fig 5B).** Moreover, compared with 100U/L of papain, expression of COX-2 and miRNA-146a were inhibited and enhanced by 200U/L of papain (p < 0.05). Similarly, 200U/L of papain combined with miRNA-146a inhibitors suppressed COX-2 expression but enhanced that of miRNA-146a compared with 100 U/L of papain combined with miRNA-146a inhibitors **(Fig 5).**

### Papain suppresses MPA-mediated expression of inflammatory mediators by upregulating miRNA-146a transcription

**Fig 6:**
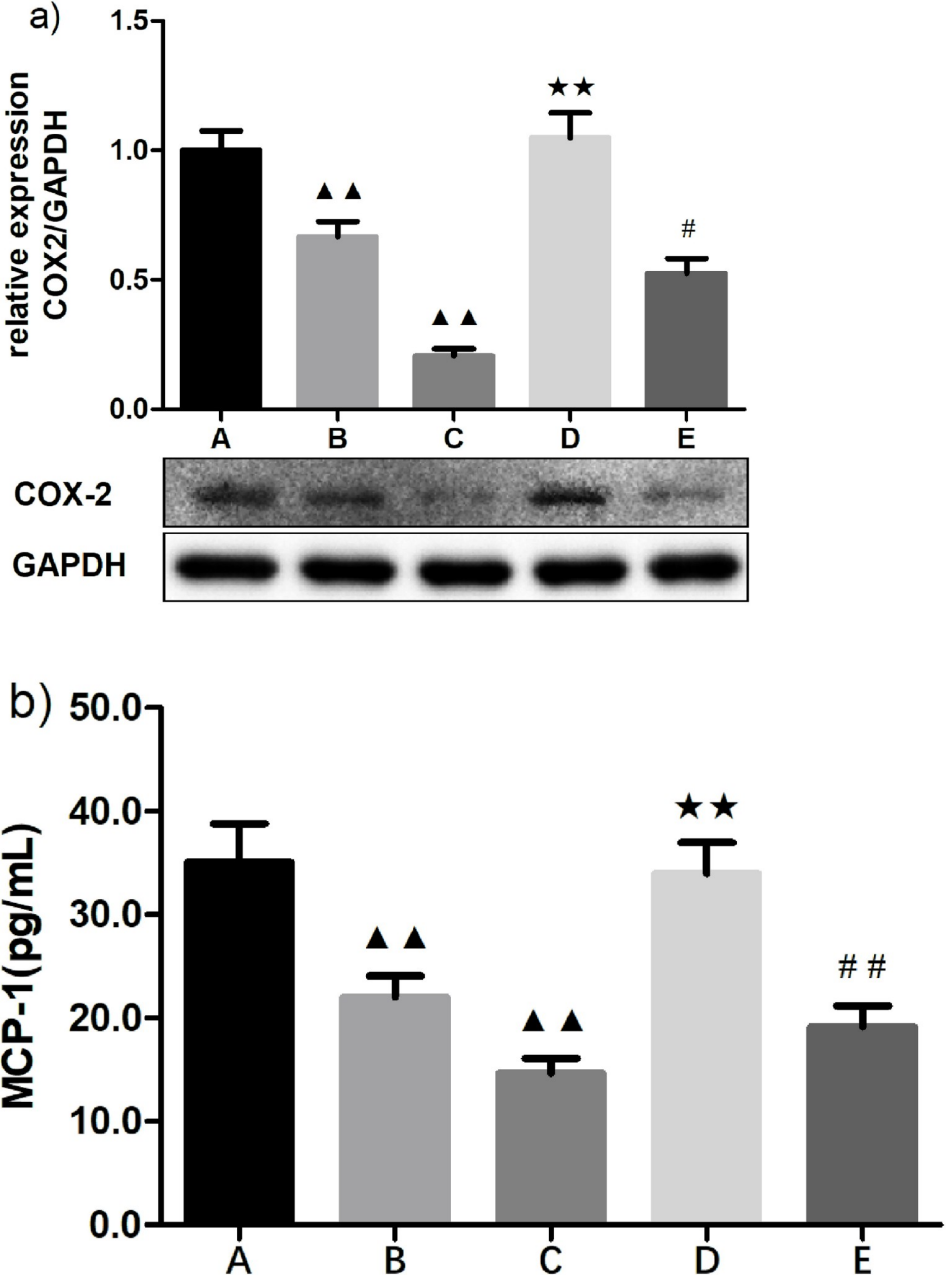
Effects of papain and miRNA-146a inhibitors on expression of COX-2 protein and MCP-1 in activated monocytes. ($\bar{x}$ ± *s*, *n* = 3 for COX-2 and *n* = 6 for MCP-1). a) Expression of COX-2 protein detected by western blot and b) MCP-1 expression was analyzed by ELISA in a co-culture system of THP-1 cells and activated platelets treated with 100 or 200 U/L of papain and miRNA-146a inhibitors, respectively. A: negative control; B: 100 U/L papain; C: 200 U/L papain; D: 100 U/L papain with miRNA-146a inhibitors; E: 200 U/L papain with miRNA-146a inhibitors. ^**#**^*P* < 0.05 compared with 200 U/L papain group; ^**▲▲, ✭✭, ##**^*P* < 0.01 compared with negative control, 100 U/L papain, and 200 U/L papain, respectively, as analyzed by LSD-*t* test.

The above results revealed that papain can upregulate miRNA-146a transcription to inhibit COX-2 mRNA expression, probably by reducing NF-κB signaling pathway activation mediated by MPA formation. Thus, we further detected expression levels of COX-2 protein and downstream MCP-1 in monocytes to evaluate the effects of papain and miRNA-146a inhibitors on inflammatory mediators. The results showed a significant decrease of COX-2 protein expression in 100 and 200 U/L of papain-treated groups (0.666 ± 0.060 and 0.205 ± 0.029 vs. 1.000 ± 0.076; p = 0.004 and 0.000, respectively) (**Fig 6A–6B** and **C** vs. **6a-A**, respectively). However, these effects exhibited an inverse tendency in the presence of miRNA-146a inhibitors (1.048 ± 0.095 and 0.525 ± 0.057 vs. 0.666 ± 0.060 and 0.205 ± 0.029; p = 0.004 and 0.042, respectively) (**Fig 6A–6D** and **6E** vs. **6A–6B** and **6C**, respectively). Moreover, downstream MCP-1 expression exhibited similar changes to that of COX-2 protein **(Fig 6B).** The powerful blocking effects of papain observed above suggest that papain can reduce COX-2 protein and subsequent MCP-1 expression to restrict inflammatory processes, primarily by upregulating miRNA-146a expression to inhibit the NF-κB signaling pathway.

## Discussion

Atherosclerosis, recognized as a chronic inflammatory disease, is correlated with excessive monocyte activation and a series of inflammatory responses [22]. Diseases related to AS feature increased plasma levels of inflammatory mediators and elevated leukocyte-platelet aggregate levels, mostly MPAs [23]. Elevation of MPAs is also an early marker of myocardial infarct and diabetes [24, 25]. Previous studies showed that interactions of monocytes and activated platelets could enhance MPA formation and subsequent monocyte activation and inflammatory responses by activating NF-κB signaling, and this process can be inhibited by NF-κB inhibitors and papain [11, 17, 21]. miRNA-146a, a transcription factor whose expression is upregulated by inflammatory factors and regulated by NF-*κ*B [25], operates in a feedback system or ‘negative regulatory loop’ to finely tune inflammatory responses by regulating NF-κB signaling [26]. However, it was unclear whether MPA formation could regulate miRNA-146a expression in activated monocytes, or whether inhibitors of MPA-mediated monocyte activation, such as papain, could inhibit NF-κB signaling by upregulating miRNA-146a expression.

In the present study, after miRNA-146a mimic- and inhibitor-transfected THP-1 cell models were successfully established, we discovered that NF-κB inhibition significantly increased the expression of miRNA-146a and had similar upregulating effects as transfection with miRNA-146a mimic. However, these effects showed opposing tendencies in the presence of miRNA-146a inhibitors, suggesting that miRNA-146a transcription might negatively regulate NF-κB pathway activation. Thus, miRNA-146a transcription may be a therapeutic target to prevent inflammatory process induced by MPA-mediated monocyte activation. In subsequent studies, miRNA-146a expression was enhanced in dose-dependent manner under conditions with papain, and miRNA-146a inhibitors could reverse the enhancing effects of papain on miRNA-146a expression. Therefore, papain might act as a potential drug for intervention of MPA formation-mediated monocyte activation and downstream inflammatory processes by regulating miRNA-146a expression. However, it remains necessary to clarify the effects of papain combined with miRNA-146a on monocyte activation and subsequent inflammatory responses after monocyte-platelet attachment.

The binding of P-selectin on platelets to leukocyte (mostly monocyte) surface PSGL-1 (P-selectin glycoprotein ligand) is an important event that enhances monocyte activation. In addition, this binding increases αMβ2 integrin (Mac-1, CD11b/CD18) expression, which stabilizes the complex and is crucial for the survival of circulating MPAs [23, 27]. Our previous study observed an obvious increase of CD11b expression in MPA-mediated monocytes [21]. In this study, we further found that percentages of CD11^+^ cells were remarkably decreased after miRNA-146a mimic transfection or NF-κB inhibitor treatment, indicating monocyte activation was inhibited by these interventions. However, miRNA-146a inhibitors augmented the percentage of CD11b^+^ cells, showing the opposite effect to miRNA-146a mimics. Therefore, this study demonstrated an inhibitory effect of miRNA-146a on monocyte activation. Our previous study confirmed the inhibitory effects of papain on monocyte activation; here, we found that papain treatment of a co-cultured system could reduce the percentage of CD11^+^ cells. Moreover, under conditions with papain and administration of miRNA-146a inhibitors, the proportion of CD11b^+^ cells nearly returned to a normal level, indicating that miRNA-146a inhibitors can indirectly reverse the inhibitory effects of papain. In addition, these results further suggest that the inhibitory effects of papain on MPA-mediated monocyte activation might be associated with upregulation of miRNA-146a expression, which may be an inhibitory mechanism of papain on NF-κB signaling.

Activated monocytes initiate the secretion of proinflammatory and atherogenic molecules [28]. Lipopolysaccharide-mediated monocyte activation promoted expression of proinflammatory factors tumor necrosis factor ⍺ and COX-2, which has essential action in the pathogenesis of AS [29]. Moreover, high levels of COX-2 expression were observed in monocytes of patients with coronary artery disease in our previous study [15]. In the progression of cardiovascular disease, Toll-like receptor 4 signaling has been recognized to promote the release of MCP-1 from activated monocytes [30]. All these studies imply that expression of COX-2 and MCP-1 may serve as important indicators of activated monocytes. In this study, transfection with miRNA-146a mimics reduced expression of COX-2 mRNA and protein, and decreased MCP-1 expression levels in monocytes activated by MPA formation. In addition, an NF-κB inhibitor showed similar inhibitory effects to a miRNA-146a mimic. However, expression of COX-2 and MCP-1 was markedly inhibited in miRNA-146a inhibitor-transfected monocytes. These results suggest that miRNA-146a transcription might reduce MPA-mediated proinflammatory responses in activated monocytes by negatively regulating NF-κB signaling. Moreover, when the co-culture system was exposure to papain, expression of miRNA-146a in monocytes was increased, and COX-2 mRNA and protein expression were accordingly downregulated in a dose-independent manner. Furthermore, as a downstream inflammatory molecule of monocyte activation, MCP-1 secretion was also reduced, consistent with the decreased expression of COX-2. These results indicate that papain promoted upregulation of miRNA-146a transcription and further ameliorated the subsequent inflammatory response involved in MPA-initiated expression of inflammatory mediators in monocytes. We further found that administration of miRNA-146a inhibitors in the co-culture system with papain reversed the protective effects of various concentrations of papain on expression of COX-2 and MCP-1, implying that selective miRNA-146a inhibition may contribute to the enhancement of inflammatory responses and diseases associated with monocyte activation. In our previous study, we revealed that papain could inhibit NF-κB activation, suggesting papain may play the same role as NF-κB inhibitors. Therefore, our findings suggest that papain inhibits MPA formation-mediated inflammatory responses in activated monocytes by reducing expression of NF-κB-regulated inflammatory mediators through upregulation of miRNA-146a transcription.

Within our knowledge, there are at least two limitations in this study. Firstly, although the NF-ĸB inhibitor BAY11-7082 was used in this study, the effect of NF-ĸB activator such as TNF-α was not observed at the same time, which might cause some indefinite conclusions, to some extent, that papain possesses the protective effects on the MPAs formation-initiated expression of inflammatory mediators by suppressing NF-ĸB signaling in THP-1 cells. However, in our previous study [21], we have found papain may play the same role of NF-ĸB inhibitor. So we did not investigate the activation of NF-ĸB. Secondly, as a digestive enzyme, papain can disrupt monocyte/platelet complexes, but this does not mean that it needs to act through miR-146a. Although we did not completely confirm the direct correlation between papain treatment and increased miRNA-146a transcription in monocytes, our study also demonstrated the papain could inhibit MPAs-mediated miRNA-146a transcription and the inflammatory response in a dose-dependent manner. However, how papain regulates the MPA-mediated expression of miR-146a in monocytes should be observed in further study.

## Conclusions

Our study suggests that papain exerts protective effects against MPA formation-initiated expression of inflammatory mediators in THP-1 cells by suppressing NF-κB signaling via upregulation of miRNA-146a transcription. It would be helpful to enrich the inhibitory mechanisms of papain on inflammation responses involved in monocyte activation induced by the interaction between platelets and monocytes, and might be useful to explore its anti-AS mechanism through further *in vitro* and *in vivo* studies.

## Supporting information

S1 Raw images (PDF)
