# Supplementary material for: Papain ameliorates monocyte-platelet aggregate formation-mediated inflammatory responses in monocytes by upregulating miRNA-146a transcription
Source: PLoS One. 2022 Nov 21;17(11):e0278059. doi: 10.1371/journal.pone.0278059 (PMC9678272; doi:10.1371/journal.pone.0278059)
Supplement: S1 Raw images — (PDF) [file pone.0278059.s001.pdf]

Figure 1 Original western blot gels for three repeats of the Figure 3 in text

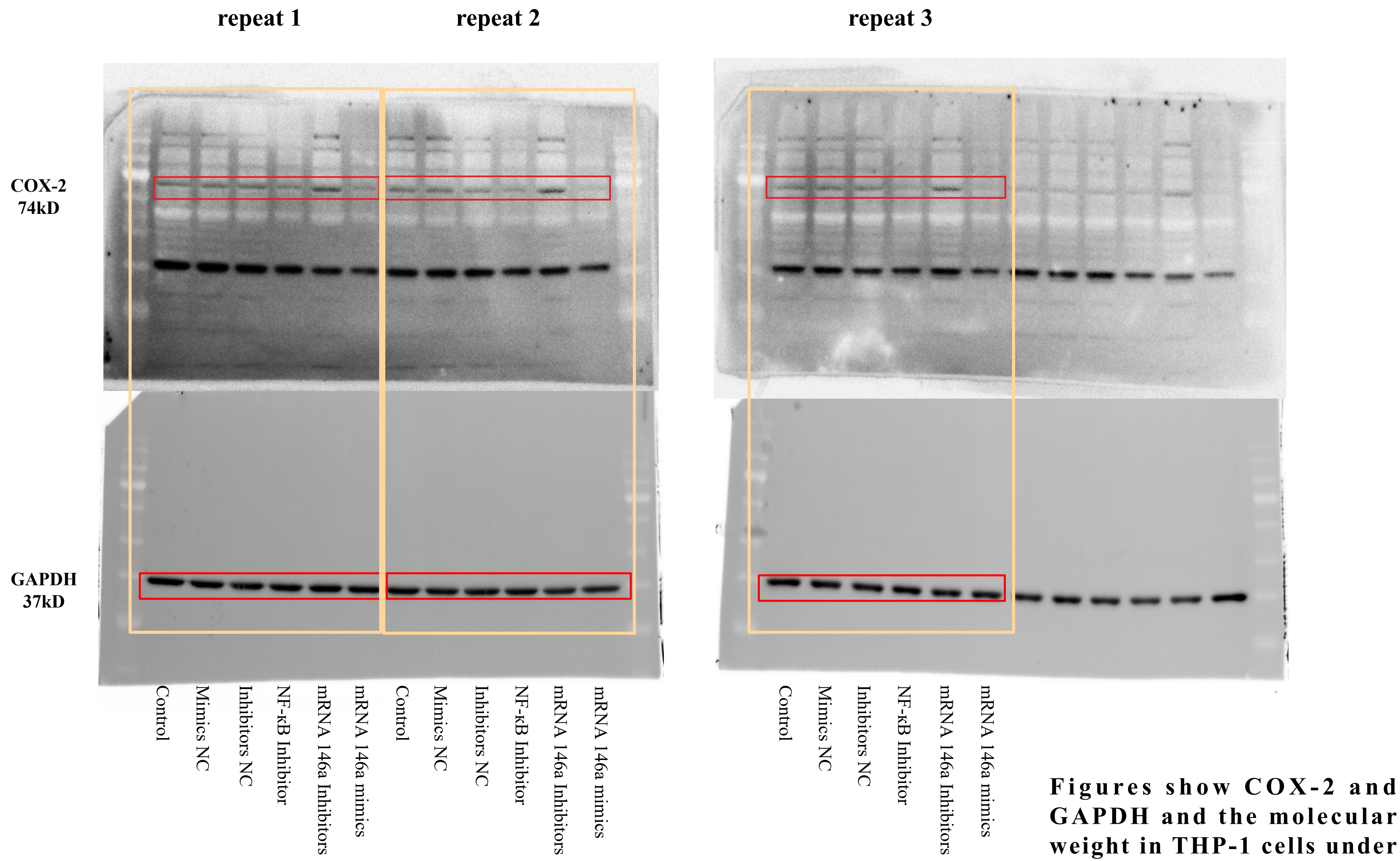

Figures show COX-2 and GAPDH and the molecular weight in THP-1 cells under different mRNA-146a treatment.

Figure 2 Original western blot gels for three repeats of the Figure 6 in text

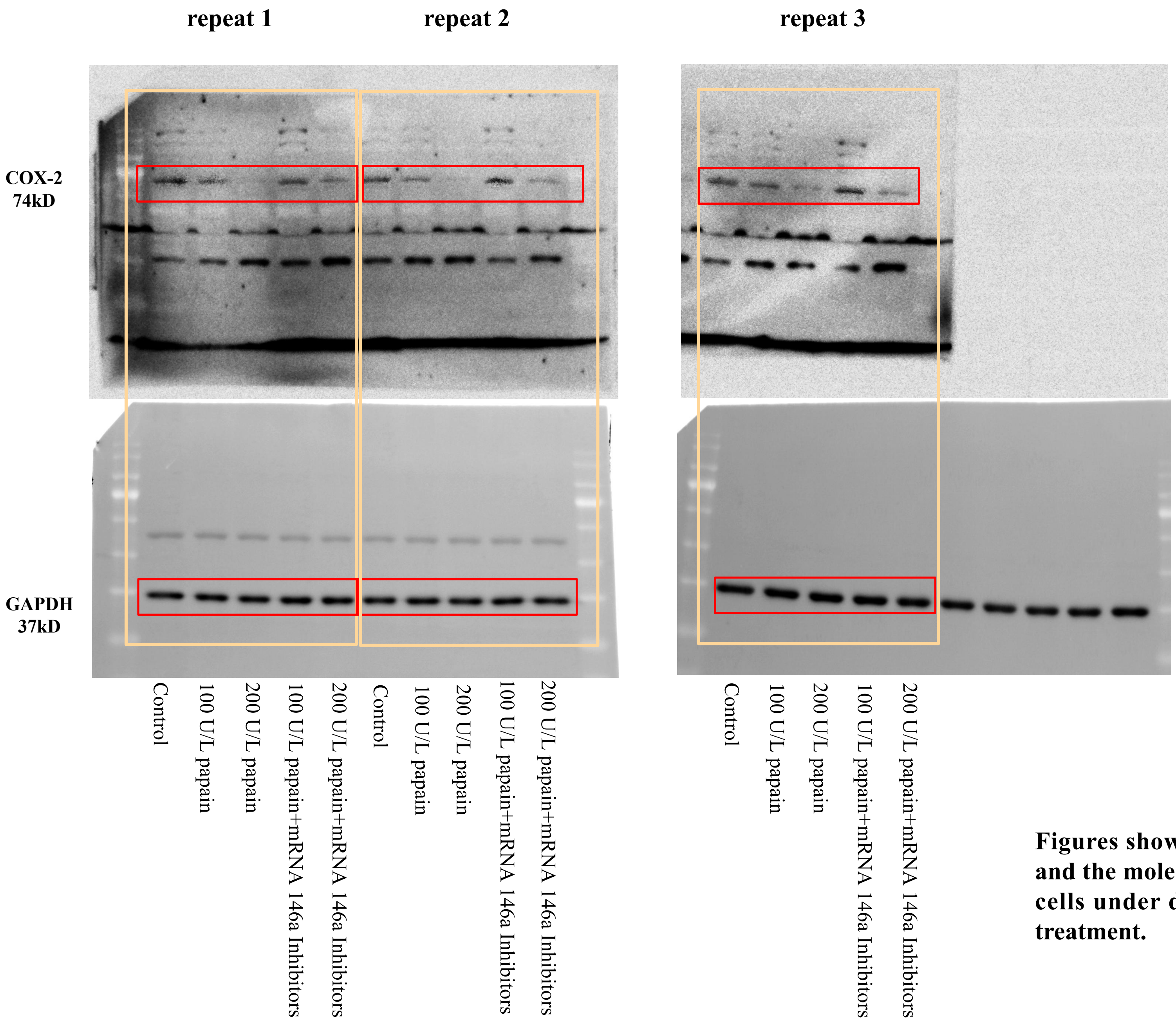

Figures show COX-2 and GAPDH and the molecular weight in THP-1 cells under different mRNA-146a treatment.
